# PB.15. An audit of our benign surgical biopsy rate in the prevalent round for the reporting year 1 April 2012 to 31 March 2013

**DOI:** 10.1186/bcr3722

**Published:** 2014-11-03

**Authors:** R Geach, E Kutt, A Valencia

**Affiliations:** 1 Bristol Royal Infirmary, Bristol, UK

## Introduction

The NHSBSP has defined standards with regard to benign surgical biopsy rates. In the prevalent population, the minimum defined standard is <3.6/1,000 with a target of <1.8/1,000. At the Avon Breast Screening Unit we have been identified as outliers.

## Methods

We retrospectively reviewed our benign surgical biopsies from the reporting year 1 April 2012 to 31 March 2013. Thirty-two patients were identified but only 16 were in the prevalent screening population. For each patient the following data were collected: mammographic abnormality; ultrasound abnormality; numerical classification (M1 to M5/U1 to U5 abnormality); lesion size (mm); type of biopsy; B3 histopathological diagnosis; and final histopathological diagnosis.

## Results

Forty-four per cent of lesions biopsied and subsequently surgically excised were microcalcifications. Seventy-five per cent of the lesions were M3 indeterminate and were small, 50% measuring <10 mm. All 16 patients had a B3 pathological diagnosis at biopsy. Forty-four per cent were AIDP, 12% sclerosing adenosis and a radial scar with the remainder other B3 diagnoses. Of the 11 patients that had AIDP reported in the initial biopsy specimen, foci of atypia were present in just four of these surgical specimens.

## Conclusion

There is inconsistent practice across the UK in the management of B3 lesions. Some may be referred for surgical excision whilst other are removed through vacuum-assisted biopsies. We perform surgical excision for any lesions with atypia. However, to reduce our benign surgical excision rate we aim to increase the number of mammotome excisions for B3 lesions without atypia.

